# Longitudinal Characterization and Biomarkers of Age and Sex Differences in the Decline of Spatial Memory

**DOI:** 10.3389/fnagi.2020.00034

**Published:** 2020-02-20

**Authors:** Marcelo Febo, Asha Rani, Brittney Yegla, Jolie Barter, Ashok Kumar, Christopher A. Wolff, Karyn Esser, Thomas C. Foster

**Affiliations:** ^1^Department of Psychiatry, McKnight Brain Institute, University of Florida, Gainesville, FL, United States; ^2^Department of Neuroscience, McKnight Brain Institute, University of Florida, Gainesville, FL, United States; ^3^Department of Physiology and Functional Genomics, Myology Institute, University of Florida, Gainesville, FL, United States; ^4^Genetics and Genomics Program, University of Florida, Gainesville, FL, United States

**Keywords:** Sexual dimorphisms, episodic memory, cognitive function, life span, longitudinal

## Abstract

The current longitudinal study examined factors (sex, physical function, response to novelty, ability to adapt to a shift in light/dark cycle, brain connectivity), which might predict the emergence of impaired memory during aging. Male and female Fisher 344 rats were tested at 6, 12, and 18 months of age. Impaired spatial memory developed in middle-age (12 months), particularly in males, and the propensity for impairment increased with advanced age. A reduced response to novelty was observed over the course of aging, which is inconsistent with cross-sectional studies. This divergence likely resulted from differences in the history of environmental enrichment/impoverishment for cross-sectional and longitudinal studies. Animals that exhibited lower level exploration of the inner region on the open field test exhibited better memory at 12 months. Furthermore, males that exhibited a longer latency to enter a novel environment at 6 months, exhibited better memory at 12 months. For females, memory at 12 months was correlated with the ability to behaviorally adapt to a shift in light/dark cycle. Functional magnetic resonance imaging of the brain, conducted at 12 months, indicated that the decline in memory was associated with altered functional connectivity within different memory systems, most notably between the hippocampus and multiple regions such as the retrosplenial cortex, thalamus, striatum, and amygdala. Overall, some factors, specifically response to novelty at an early age and the capacity to adapt to shifts in light cycle, predicted spatial memory in middle-age, and spatial memory is associated with corresponding changes in brain connectivity. We discuss similarities and differences related to previous longitudinal and cross-sectional studies, as well as the role of sex differences in providing a theoretical framework to guide future longitudinal research on the trajectory of cognitive decline. In addition to demonstrating the power of longitudinal studies, these data highlight the importance of middle-age for identifying potential predictive indicators of sexual dimorphism in the trajectory in brain and cognitive aging.

## Introduction

Variability in cognitive function with age is due, in part, to a lifetime of differences in experiences. Compared to cross-sectional studies, longitudinal studies provide better characterization of the onset of brain aging and cognitive decline (Nyberg et al., 2012). In addition, measures obtained at several points in time provide an assessment of individual differences in the rate of change in cognition and biomarkers of aging (Wilson et al., 2002; Du et al., 2006; Driscoll et al., 2009; Pudas et al., 2013, 2014; Zahodne et al., 2013; Pfefferbaum and Sullivan, 2015; Rajan et al., 2015; Staffaroni et al., 2018; Vidal-Pineiro et al., 2018). Recent recommendations from the third Cognitive Aging Summit included longitudinal studies of neuroadaptive processes over the lifespan in shorter-lived animal models (Wagster and King, 2019). Longitudinal studies in animals have several advantages for examining cognitive aging. In the case of rodent models, there is considerable knowledge about cognitive aging, and the shorter lifespan facilitates examination of interventions. Relative to cross-sectional studies, longitudinal testing is able to control for batch effects and guard against problems associated with the history of experience (Sabolek et al., 2004). For example, impairments may arise due to an interaction of age and the length of exposure to social isolation or an impoverished environment (Winocur, 1998; Bell et al., 2009; Diniz et al., 2010; Volkers and Scherder, 2011; Sampedro-Piquero et al., 2014; Diamond, 2018; Sparling et al., 2018; Wang et al., 2018). In turn, longitudinal studies need to control for influences that carryover from one testing situation to another. Carryover effects include memory for procedural aspects of the task (Dellu et al., 1997; Guidi et al., 2014). Thus, procedural memories acquired in youth are resistant to age-related forgetting, such that aged animals are better able to perform a spatial reference memory task on the water maze relative to when they were first tested as young adults (Algeri et al., 1991; Pitsikas et al., 1991; Gyger et al., 1992; Dellu et al., 1997; Vallee et al., 1999; Markowska and Savonenko, 2002; van Groen et al., 2002; Vicens et al., 2002). In contrast, many tasks that involve repeated acquisition of rapidly acquired and flexible spatial information (e.g., spatial episodic or working memory) exhibit minimal carryover effects and longitudinal studies indicate that, similar to humans (Nyberg and Pudas, 2019), impaired memory emerges around middle-age in rats and mice (Ando and Ohashi, 1991; Forster and Lal, 1992; Vallee et al., 1999; Hartman et al., 2001; Markowska and Savonenko, 2002; Dellu-Hagedorn et al., 2004; Sabolek et al., 2004).

Longitudinal studies that address sex differences in older humans generally indicate poorer episodic memory in men. There is a debate concerning sex differences in the trajectory of cognitive impairments, with results indicating no difference or a steeper decline in males (Finkel et al., 2003; Ferreira et al., 2014; Lundervold et al., 2014; McCarrey et al., 2016; Olaya et al., 2017; Hughes et al., 2018; Lee et al., 2018; Casaletto et al., 2019). Cross-sectional rodent studies indicate age and sex differences in tasks that involve repeated acquisition of spatial information and episodic memory (Lukoyanov et al., 1999; Markowska, 1999; Rossetti et al., 2018); nevertheless, most longitudinal studies in animal models of aging have not compared sex differences in the decline of memory.

The current study was designed to examine the age-related decline of memory in relation to other behavioral and psychological measures and determine the relationship between these measures and biologically relevant variables, including sex, circadian adaptability, and brain functional connectivity. Studies employing magnetic resonance imaging in animal models have emerged as a valuable strategy for characterizing the relationship between cognitive function and neural circuits that underlie the cognitive process of interest (Liang et al., 2015; Ash et al., 2016). Resting state connectivity provides information on intrinsic functional brain organization, and similar resting state functional connectivity is observed between humans and animal models, including rodents, rabbits, and monkeys (Febo and Foster, 2016). The analysis of brain wide resting state functional connectivity in the rodent brain is facilitated by the use of network science algorithms, which provide insight on the topology of connectivity patterns under normal and disease states (Diaz-Parra et al., 2017).

## Experimental Procedures

### Subjects

Male (5 months, *n* = 10) and female (5 months, *n* = 10) Fisher 344 rats were obtained from the National Institute on Aging colony (Taconic) through the University of Florida Animal Care and Service facility. Animals were pair housed (2 per cage) with *ad libitum* access to water and food. All procedures involving animal subjects were approved by the Institutional Animal Care and Use Committee, and were in accordance with guidelines established by the United States Public Health Service Policy on Humane Care and Use of Laboratory Animals.

### Behavioral Testing Procedures

#### Timeline

The rats underwent the following behavioral assessments every 6 months, starting at 6 months of age. For each testing period (6, 12, 18 months), physical measures were acquired during week 1–2 of testing, in the following order, grip strength, Rotarod, 24 h wheel running activity, with 24–48 h between tests. During week 2–3, the animals were tested for response to an open field, followed 24–48 h later by measures of the latency to enter a novel environment. During weeks 3–5, animals were tested on the water maze, followed 3 days later by novel location recognition testing. In order to ensure that animals acquired the procedural aspect of the water maze prior to spatial testing, animals were trained on a 1 day cue discrimination version at 6 months (Guidi and Foster, 2012; Guidi et al., 2014). Due to the perseverance of procedural memory, the cued version of the task was omitted for months 12 and 18. Three days after cue discrimination testing, animals received three consecutive days testing (one trial per day) on the delayed-matching-to-place (DMTP) task, with a one minute delay between the acquisition and retention trials. This was followed by 9 consecutive days of testing (one trial per day) on the DMTP task with variable delays (5, 30, 120 min). The next week, animals began 4 weeks in cages to determine the circadian parameter, activity onset, and to test for the number of days required to re-entrain activity onset following an acute 6 h phase advance. At the 6-month testing time, rats were initially housed singly in wheel cages to assess activity onset and daily wheel activity. However, the animals did not use the wheels so baseline circadian activity onset could not be determined. Therefore, starting at 12 months, rats were singly housed in cages that were equipped with an infra-red motion detector to monitor activity onset before challenging the rats with a 6 h phase advance. Following the 12 months battery of testing, animals were aging pair housed and permitted 5 weeks to readjust to the normal light:dark schedule before functional magnetic resonance brain imaging.

### Physical Measures

#### Grip Strength Test

Forelimb grip strength was determined using an automated grip strength meter (Columbus Instruments, Columbus, OH, United States) as previously described (Cui et al., 2009; Carter et al., 2011; Zhou et al., 2011; Kumar et al., 2012). The mean force (grams) was calculated over three trials, separated by 2–4 min.

#### Rotarod Performance Test

Animals were placed on a horizontally oriented, rotating cylinder (Rotarod) suspended above a cage floor. On day 1, animals were familiarized to the Rotarod by placing them on the rod for 2–3 min. On day 2, animals were presented with three trials, with each trial separated by 10 min. For each trial, the Rotarod was initially set at a speed of 1 rpm and increased by 1 rpm/10 s for 2 min. The latency of time to fall off the Rotarod was averaged across the three trials.

#### Wheel-Running Activity

Starting at 10 am (lights on 6 am:lights off 6 pm), animals were individually housed in cages equipped with activity wheels (1.068-m circumference, Fisher Scientific, Pittsburgh, PA, United States). Each wheel was equipped with a magnetic switch and counter to record the number of wheel revolutions over a 24 h period. Animals fed *ad libitum* tend to decrease their running activity during aging and a slight food restriction prevents this decline (Cui et al., 2009; Kumar et al., 2012). Therefore, animals had free access to water but not food for the 24 h of confinement to the wheel-running cage. The total number of wheel revolutions were recorded, as well as the number that occurred during the lights off period (6 pm to 6 am). The total number of wheel revolutions over the 24 h, as well as the number that occurred during the lights off period (6 pm to 6 am) was converted to meters for statistical analysis.

### Response to Novelty

#### Open Field

Rats were placed in an open plexiglass arena (23 in × 23 in) for 20 min and were recorded using EthoVision XT 6.0 for total distance traversed, velocity, and percent time in the inner region of the arena.

#### Response to a Novel Environment

Novelty testing was conducted as previously described (Lukkes et al., 2016). Briefly, rats were placed in one of two connected chambers (9.75 in × 9.75 in each) for 20 min a day for three consecutive days to permit for habituation. Forty-eight hours after the last habituation session, rats were placed in the habituated chamber and a door permitting access to the adjacent chamber was opened. As a measure of novelty seeking, the latency to enter the adjacent room (both front paws across the threshold) was recorded, as well as the amount of time spent in the novel chamber for a 20 min period. Each chamber was distinct, in terms of wall patterns (i.e., circles vs. stripes; diamonds vs. hexagons, etc.) and flooring (bars vs. black plexiglass) to provide a novel exploratory experience. The initial chamber selected for habituation was counterbalanced across rats.

### Cognitive Measures

#### Water Maze Testing for Memory

##### Apparatus

Methods for training animals on the water maze task have been adapted from Guidi et al. (2015). Animals were trained in a black tank, 1.7 m in diameter, positioned in a well-lit room containing (when appropriate) an assortment of 2- and 3-dimensional cues. Water (27 ± 2°C) was maintained at a level approximately 8 cm below the surface of the tank. Across all water maze tasks, training and probe trials were limited to 60 s.

##### Cue discrimination training

Deficits in spatial learning on the water maze can result from a fear to move away from the wall (thigmotaxis). In order to ensure that all animals acquired the procedures (i.e., how to swim and the wall is not a means of escape), rats were first trained on the cue discrimination version of the water escape task (Foster et al., 2012). Animals were habituated to the pool by allowing for a 30 s free swim and 4-guided attempts to climb onto the escape platform from the four different cardinal directions. The platform was extended approximately 1 cm above the water level and a white polystyrene flag was attached. Training consisted of five blocks comprised of three trials with all training massed into one day. Inter-trial intervals were 30 s and inter-block intervals were approximately 15 min. For each trial, a rat was placed in the water in one of four equally spaced start locations (N, S, E, and W) and was allowed 60 s to escape onto the platform. If an animal did not escape the water maze within the allotted time, the rat was gently guided to the platform. Rats remained on the platform between trials. After each trial block, the rats were placed in home cages under warming fans in order to prevent hypothermia. The platform position and start locations were randomized and relocated prior to the start of each subsequent trial. The distance traveled to find the platform was recorded and averaged for each block. The procedural aspects of the swim task are well retained over the course of aging; therefore, this task was only performed once, at 6 months.

##### Delayed-matching-to-place (DMTP) task

Three days following the initial cue discrimination training, animals were trained on a modified version of the DMTP task, which is sensitive to aging (Foster, 2012; Guidi et al., 2015). The platform was lowered to just beneath the water surface and animals were trained to find a hidden platform. Each training session consisted of two trials per day, an information trial and retention trial, in which the platform remained in the same position. Between days, the platform position varied in a semi-random manner. The start position varied and was always distal from the platform. Following the information trial, rats remained on the platform for 30 s and then were moved to a holding chamber under warm air during the inter-trial interval (ITI) delay. For the first 3 days of the task, a 60 s ITI was imposed between the trials to acclimate the rats to the spatial working memory task procedures. During the next nine days of testing, the ITI between the information trial and the retention trial was randomly varied between 5, 30, or 120 min, such that each delay occurred three times. The distance traveled to find the platform was recorded for each trial and a savings score was calculated as the difference between the initial information trial and subsequent retention trial (e.g., distance trial 1–distance trial 2). For each delay, the savings scores were averaged for 3 days of testing.

#### Novel Location Recognition

Test objects were presented in an open plexiglass arena (23 in × 23 in). The objects were made of ceramic and glass with dimensions of 6.5 × 6.5 × 11.5 cm (l × w × h) and shaped to look like animals (e.g., birds, fish). The rats’ behavior in the arena was monitored by an overhead video recorder and scored by two observers (verified by inter-rater reliability testing). The test session was divided into a sample phase and a test phase, 2 h later. In the sample phase, two identical objects (A1 and A2) were placed in two adjacent corners of the arena approximately 10 cm from the edges. The rat was then placed in the arena so that it faced away from the objects. The rat was allowed to explore the arena and objects for 5 min and then returned to its home cage in the testing room where it remained for the 2 h interval. During the test phase, one object was moved to a different corner and the time exploring the objects was recorded for 5 min. Different objects and location placement was used at 6, 12, and 18 months. All objects and the arena were thoroughly cleaned with 70% ethanol between trials to remove odors.

### Acute Phase Advance Testing

The rat cages were housed in circadian cabinets (Actimetrics) that allow for precise light control. Lighting of the cabinets and real time data collection from the infra-red devices (beam break counts) were controlled and collected with ClockLab software. At the 6-month testing time, rats were initially housed singly in wheel (0.54 m circumference) cages to record activity. However, due to the relatively small size of the wheel relative to the size of the rats, the animals exhibited little baseline wheel activity. Therefore, the animals were removed from the cages before acute phase advance testing. Starting at 12 months, rats were singly housed in cages equipped with an infra-red motion detector to monitor cage activity over 24 h/day. After the 2-week acclimation period in the cages with 12 h light:12 h dark, the rats were exposed to one bout of a 6-hour advance in the time for lights-off and then they were maintained on the new 12 h light: 12 h dark for 2 weeks to record the time it took for each rat to entrain to the new light:dark schedule. The threshold to define activity onset was set at five counts of activity/minute for a period of the first 5 min following lights off. The rats were considered re-entrained to the new light:dak schedule when the rats became active in the cage within 10 min of lights off. During the course of the study, body weight and cage mobility were examined weekly.

### Statistical Analysis for Behavior

Analyses of variances (ANOVAs) were repeated across age and included sex as a relevant variable. Subsequent ANOVAs were performed within each age to localize sex differences and repeated across age for each sex to localize age differences for each sex. Fischer’s LSD tests were used for *post hoc* comparisons (*p* < 0.05). Pearson’s multiple regressions, including power analysis (1-β), were performed to examine the relationship between the averaged savings scores for 12 months DMTP task, sex, and other behavioral measures.

### Functional Magnetic Resonance Imaging (fMRI)

#### fMRI Acquisition

Following completion of behavioral testing at 12 months, animals were prepared for imaging. Rats were imaged under 1.5% isoflurane gas anesthesia delivered at 1.5 ml/min in medical grade air (Liu et al., 2011, 2013). Spontaneous breathing rates were continuously monitored during setup and during MRI scanning (SA Instruments). A water recirculation system was used to maintain a core body temperature at 37–38°C (Gaymar). Images were collected on a 4.7 T/33 cm horizontal magnet (Magnex Scientific) with an 11.5-cm-diameter gradient insert (670 mT/m maximum gradient strength at 300 Amps and a 120 μs rise time; Resonance Research Inc.) and controlled by VnmrJ 3.1 software (Agilent). For B1 field excitation and radio frequency (RF) signal detection, a quadrature transmit/receive RF coil tuned to 200.6 MHz 1 H resonance was used (Air MRI, Holden, MA, United States). Functional image series were collected using a two-shot spin-echo echoplanar imaging (EPI) sequence with the following parameters: echo time (TE) = 50 ms; repetition time (TR) = 1 s; 32.5 × 32.5 mm in-plane; 12 slices with 1.5 mm thickness per slice; data matrix = 64 × 64 was used. A total of 210 repetitions were collected per each 7 min EPI scan, with two scans acquired per rat. No stimuli were presented during functional scanning. High resolution T2 weighted fast spin echo images were collected for image overlay and reference-to-atlas registration (TE = 45 ms; TR = 2 s; echo train length = 8; number of averages = 10; data matrix = 256 × 256).

#### Image Processing and Statistical Analysis

Brain masks were manually created using high-resolution anatomical scans to remove non-brain voxels on itkSNAP^1^. The FMRIB Software Library linear registration program *FLIRT* (Jenkinson et al., 2002) was used to align cropped brain images to a rat brain template. For each subject, registration matrices were saved and used to transform functional datasets into an atlas space for preprocessing and analysis. Over the series of 210 images, slight displacements in individual images and slice timing delays were corrected. In addition, time-series spikes were removed using Analysis of Functional NeuroImages (AFNI; Cox, 1996). Linear and quadratic detrending, spatial blurring (1.1 mm FWHM), and intensity normalization were applied to all images. Based on their location in the segmented atlas, cerebroventricular and white matter signals were extracted and used as nuisance regressors for removal from datasets. Brain signals that contain higher frequency oscillations were removed by a voxelwise temporal bandpass filter (between 0.01 and 0.1 Hz) before time-series correlation analyses were performed.

Based on the atlas-guided seed location, time series fMRI signals were extracted per each region of interest (ROI) from a total of 75 ROI in the left and right hemispheres (150 total voxel seed locations). The first nine images in each functional time series were not used to avoid unstable fMRI signal intensity variations that are typically found in the initial images. Voxelwise cross-correlations were conducted to create correlation coefficient (Pearson r) maps (Colon-Perez et al., 2016) and the Pearson r coefficients were then subjected to a voxelwise *z*-transformation. Pearson r coefficients were exported for seed-based functional connectivity and network analyses in MATLAB (MathWorks). AFNI was used to generate composite functional connectivity maps for cortical and subcortical seed regions.

Next, we calculated basic graph theory metrics to assess the topology of functional connectivity networks. The Brain Connectivity Toolbox for MATLAB was used to analyze resting-state fMRI data (Rubinov and Sporns, 2010). Symmetric adjacency matrices were organized in MATLAB [graph size = *n*(*n*−1)/2, where n is the number of vertices or nodes represented in the graph as 150 ROIs]. Matrix *z*-values were used as edge weights and these were normalized such that all matrices entries had edge weight values that ranged from 0 to 1. Node self-connections were omitted and set to 0 along the matrix diagonal. All graph theory metrics were calculated for several thresholds that preserved the top strongest functional connectivity *z* values per graph (e.g., edge density values of 5–50%). Node strength (the sum of edge weights), characteristic path length (the potential for communication between pairs of structures), assortativity (an index of the tendency of nodes to group according to their degree), clustering coefficient (the degree to which nodes cluster together in groups), modularity (the degree to which the network may be subdivided into clearly delineated groups or communities), and small world coefficient (the degree to which functional brain networks deviate from randomly connected networks) were calculated for weighted and unweighted graphs of rat brain functional connectivity graphs and their corresponding randomized connectivity graphs (Newman, 2003; Newman and Girvan, 2004; Boccaletti et al., 2006; Saramaki et al., 2007; Humphries and Gurney, 2008). A summary of the above metrics and their operational definitions have been previously reported (Rubinov and Sporns, 2010). These were also calculated for random (“null”) networks as a comparison.

The small world coefficient was calculated in each rat by comparing their functional connectivity graph to a randomized version of the same graph (Watts and Strogatz, 1998). Edge weights were randomly reassigned to different node pairs using a total of 10 random swaps to generate a random graph that preserved the original strength, density, and degree distributions. The clustering coefficient was calculated for the rat functional connectivity graphs and this was divided by the clustering coefficient for the randomized graph to give a normalized clustering coefficient value (gamma = CC_rat–fMRI_/CC_random_). Similarly, characteristic path length for rat functional connectivity graphs was divided by the characteristic path length of the randomized graph to give a normalized value (lambda = PL_rat–fMRI_/PL_random_). The small world coefficient per rat was then calculated as gamma/lambda, with small world values of 1 representing random networks and values above 1 typical of real networks (Erdös and Rényi, 1960; Humphries and Gurney, 2008). Brain-Net viewer was used to generate brain networks (Xia et al., 2013). An average matrix was generated for each experimental group (male or female and poor or best performers). Thresholding for these was set at an edge density of 10%, and a minimum spanning tree algorithm was applied to visualize the largest connected component per group (Hagmann et al., 2008). In these rat brain connectomes, the node size and color were scaled by the strength of the node, while edges were scaled by *z* scores above 0.2.

For statistical analysis of network connectivity, we first compared the mean values of the network connectivity metrics between male vs. female rats using an unpaired two-tailed *t*-test, assuming heteroscedastic variances. In a separate analysis, we re-grouped datasets into poor vs. best performers as indexed by overall performance on the DMTP task. The savings scores recorded for the DMTP task at 12 months were averaged across all delays and a mean split of averaged savings scores was employed to classify animals as poor or best performers at 12 months. In addition to the analysis of network metrics, we also carried out between-groups statistical comparisons on seed-based functional connectivity maps using statistical programs in AFNI. The resultant statistical maps were set at a threshold of *p* < 0.05. Network measures are summarized in Supplementary Figures S1, S2.

## Results

Three females and one male were removed due to development of health issues following the 12 months of behavioral testing. These four animals were not included in the repeated measures analyses. However, since animals were healthy at 12 months, these animals were included in fMRI studies and multiple regressions of cognitive function at 12 months.

### Weight, Grip Strength, Sensorimotor Function, Activity Measures

#### Weight

**FIGURE 1:**
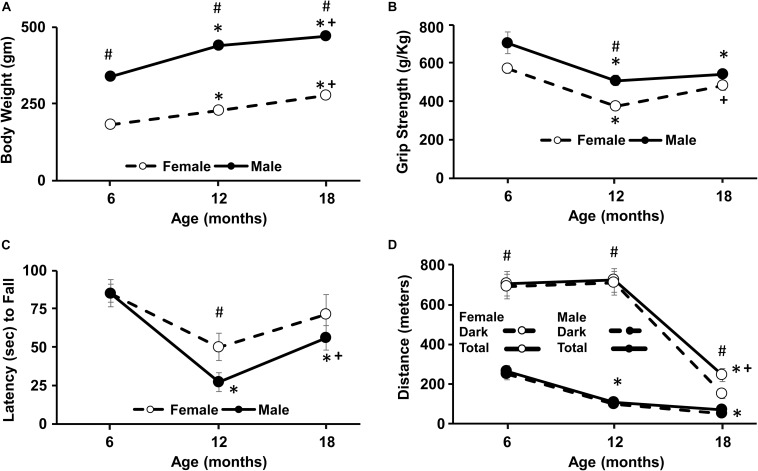
Longitudinal change in weight, grip strength, Rotarod, and wheel-running activity. For this and all subsequent figures, filled circles represent males and open circles represent females, and each point represents the mean ± SEM. **(A)** Body weight increased with age and was greater for males. **(B)** Grip strength declined between 6 and 12 months for both sexes and was greater in males at 12 months. **(C)** Latency to fall on the Rotarod decreased from 6 to 12 months in both sexes and a sex difference was evident at 12 months. **(D)** For wheel running activity, the total distance over the 24 h period (solid line) was mainly due to activity occurring during the lights off period (dashed lines). Females were consistently more active than males, exhibiting an increase in total activity at each age. For males, the greatest decline in activity occurred from 6 to 12 months and from 12 to 18 months in females. Pound sign indicates a significant (*p* < 0.05) sex difference at each age. For each sex, asterisks indicated a significant (*p* < 0.05) difference from 6 months and + sign indicates a difference between 12 and 18 months.

A repeated measures ANOVA across age for weight indicated an interaction of sex and age [*F*(2,28) = 21.2, *p* < 0.0001]. *Post hoc* tests indicated males were consistently heavier than females at each time point. Repeated measures ANOVAs within each sex indicated a significant (*p* < 0.0001) increase in weight for both sexes and *post hoc* test indicated that the weight for each time point was different from the other time points (Figure 1A).

#### Grip Strength Test

A repeated measures ANOVA indicated a main effect of sex [*F*(1,14) = 12.4, *p* < 0.005] with greater strength for males and an effect of age [*F*(2,28) = 23.2, *p* < 0.0001] in the absence of an interaction. *Post hoc* analysis to localize sex differences indicated that females exhibited reduced grip strength relative to males at 12 months. Repeated measures ANOVAs indicated an age effect within each sex (*p* < 0.001) and *post hoc* tests indicated that grip strength declined for males at 12 and 18 months relative to 6 months. For females, a decline from 6 to 12 months was followed by a significant increase from 12 to 18 months (Figure 1B).

#### RotaRod

The time that animals remained on the RotaRod decreased with age [*F*(2,28) = 13.6, *p* < 0.0001] with a trend (*p* = 0.09) for a difference due to sex. *Post hoc* analysis localized the sex difference to 12 months, with a shorter latency to fall for males. Repeated measures ANOVAs within each sex indicated a significant effect of age only for males [*F*(2,16) = 15.97, *p* < 0.0005] and *post hoc* analysis indicated a decline in fall latency at 12 and 18 months relative to 6 months and a significant increase from 12 to 18 months (Figure 1C).

#### Activity Wheel

During the 24 h access to the running wheel, most of the activity occurred during the dark period. Total activity exhibited a sex by age interaction [*F*(2,28) = 26.4, *p* < 0.0001] with females being more active than males at every time point. Repeated measures ANOVAs indicated an age effect within each sex (*p* < 0.001) and *post hoc* tests indicated that males exhibited a decline in total 24 h activity at 12 and 18 months relative to 6 months. In contrast, females exhibited a decline at 18 months, relative to 6 and 12 months (Figure 1D).

### Response to Novelty

#### Response to Novelty

**FIGURE 2:**
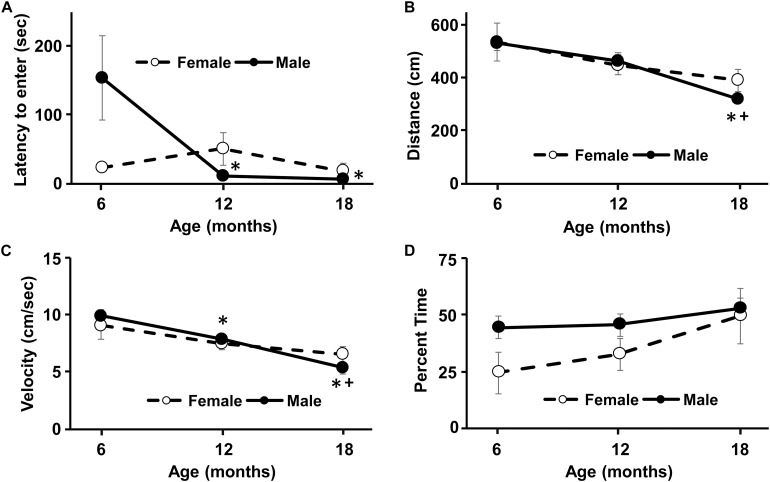
Longitudinal change in response to a novel environment and activity in an open field. No sex difference was observed for any of the measures. **(A)** An age-related decrease in the latency to enter the novel environment was due to males, such that the latency to enter the novel environment decreased at 12 and 18 months compared to 6 months. Similarly, for the open field, the distance traveled **(B)** and the velocity of movement **(C)** decreased with age, due mainly to performance by males. **(D)** No age or sex difference was observed for the percent time in the inner region of the open field. Asterisks indicated a significant (*p* < 0.05) difference from 6 months and + sign indicates a difference between 12 and 18 months for males.

A repeated measures ANOVA for latency to enter the novel environment exhibited a sex by age interaction [*F*(2,28) = 5.0, *p* < 0.05]. *Post hoc* tests could not localize the sex difference and repeated ANOVAs for each sex indicated an age effect only for males [*F*(2,16) = 5.87, *p* < 0.05]. *Post hoc* tests indicated that the latency to enter the novel environment decreased at 12 and 18 months compared to 6 months for males (Figure 2A). No age or sex difference was observed for the time spent in the novel chamber.

#### Open Field

A repeated measures ANOVA indicated an age-related decrease in distance traveled [*F*(2,28) = 11.3, *p* < 0.0005], in the absence of a sex difference. Repeated measures ANOVAs for each sex indicated an age effect only for males [*F*(2,16) = 12.92, *p* < 0.0005]. *Post hoc* test indicated that males decreased their distance in the open field at 18 months relative to 6 and 12 months (Figure 2B). A repeated measures ANOVA indicated an age-related decrease in movement velocity [*F*(2,28) = 17.0, *p* < 0.0001], in the absence of a sex difference. Repeated ANOVAs for each sex indicated an age effect only for males [*F*(2,28) = 25.05, *p* < 0.0001]. *Post hoc* test indicated that males decreased velocity in the open field from 6 to 12 months and at 18 months relative to 6 and 12 months (Figure 2C). No effect of sex or age was observed for the percent time spent in the inner region of the arena (Figure 2D).

### Cognitive Measures

#### Spatial Memory on the DMTP Task

**FIGURE 3:**
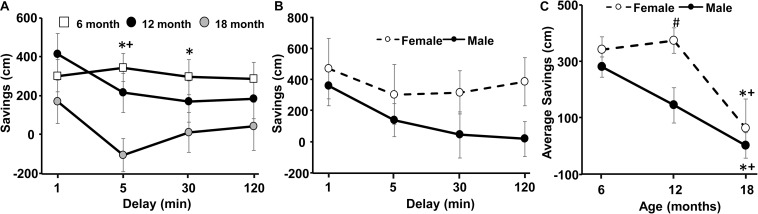
Decline in spatial memory with age. **(A)** Age- and delay-related decline in savings scores for delays of 1–120 min at the three ages. **(B)** A sex difference at 12 months was due to better performance by females. **(C)** Savings scores, averaged across each delay, for each animal, and plotted for the three different ages. Pound sign indicates a significant (*p* < 0.05) sex difference at each age. Asterisks indicated a significant (*p* < 0.05) difference from 6 months and + sign indicates a significant difference (*p* < 0.05) between 12 and 18 months.

An ANOVA repeated across ages (6, 12, 18 months) and delay times (1, 5, 30, 120 min) indicated a main effect of sex [*F*(1,14) = 5.56, *p* < 0.05] and an interaction of age and delay [*F*(2,3) = 3.43, *p* < 0.005]. *Post hoc* ANOVAs across delays, within each age indicated that savings scores declined at 18 months relative to 6 months for the 30 min delay and at 18 months relative to 6 and 12 months for the 5 min delay. Figure 3A illustrates the decrease in savings scores with advancing age and longer delays. Furthermore, a one-tailed *t*-test indicated that, at 6 months, savings scores for all delays were above chance (i.e., savings score of 0). At 18 months all savings scores were not different from chance. For the 12 months test, all savings scores were above chance, except for the 30 min delay. ANOVAs repeated across delays within each age group localized a sex difference to 12 months [*F*(1,14) = 7.8, *p* < 0.05], with better performance by females (Figure 3B). *Post hoc* tests failed to localize the sex difference to a specific delay. To follow-up on the sex differences and memory performance across age, we averaged the savings scores across all delays for each animal at each age (Figure 3C). A repeated measures ANOVA confirmed an effect of sex [*F*(1,14) = 5.56, *p* < 0.05] and age [*F*(2,28) = 12.77, *p* < 0.0001] in the absence of an interaction. *Post hoc* analysis localized the sex difference to 12 months, with decreased savings scores for males. Repeated measures ANOVAs within each sex indicated a significant effect of age for each sex (*p* < 0.05) and *post hoc* analysis indicated a decline savings score at 18 months relative to 6 and 12 months within each sex.

#### Novel Location Recognition

**FIGURE 4:**
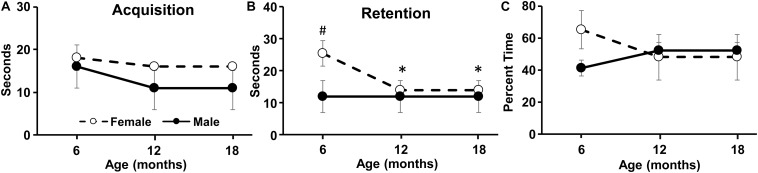
Longitudinal change in the novel location task. The graphs indicate the time spent exploring the objects during the **(A)** acquisition phase, **(B)** retention phase, and **(C)** the percent time exploring the object in the novel location during retention testing. Pound sign indicates a significant (*p* < 0.05) sex difference. Asterisks indicated a significant (*p* < 0.05) difference from 6 months for females.

No age or sex difference was observed for the time spent exploring objects during the acquisition phase (Figure 4A). For the 2 h test phase, there was a significant [*F*(2,28) = 3.6, *p* < 0.05] decline in exploration time for both objects over the course of aging and an interaction of sex and age [*F*(2,28) = 3.5, *p* < 0.05]. *Post hoc* tests indicated a sex difference at 6 months with more exploration during the test phase by females (Figure 4B). Repeated ANOVAs for age effects on the test phase exploration for each sex indicated a tendency (*p* = 0.07) for an age effect in females, and *post hoc* tests localized a decrease in the 2 h exploration at 12 and 18 months relative to 6 months (Figure 4B). No age or sex difference was observed for the percent time exploring the novel location and the exploration time was not different from chance (i.e., 50%) (Figure 4C).

### Circadian Adaptation

**FIGURE 5:**
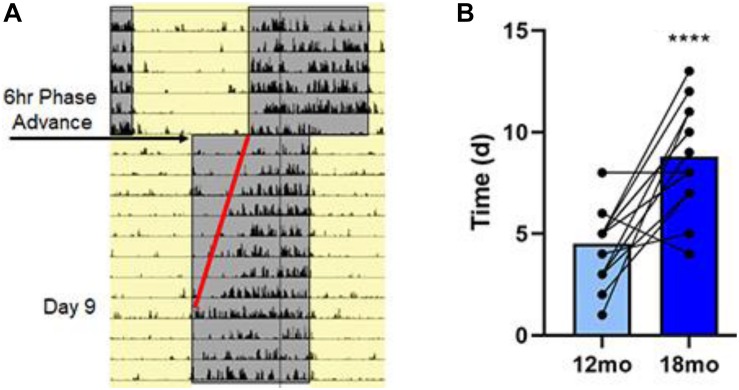
Aging is associated with lengthening in the time to entrain to a shift in light/dark cycle. **(A)** Representative actogram for an 18-month-old rat following a single 6 h phase advance. Cage activity was monitored by infra-red motion detecting and analysis of activity onset was determined; this rat took 9 days to establish activity onset with lights off as highlighted by the red line. **(B)** Summary data for the time (in days) required to entrain to the 6 h phase advance for all the rats at 12 and 18 months. Average data and individual rat data for both ages demonstrating that there was an age-associated lengthening in the time to entrain for the 18-month rats. Asterisks indicated a significant (*p* < 0.0001) difference from 12 month olds.

Examination of the ability of the rats to re-entrain to a new light:dark cycle was not completed at 6 months due to insufficient baseline wheel activity. Starting at 12 months, the rats were singly housed in cages equipped with an infra-red motion detector to monitor cage activity over 24 h/day. For female animals, the data were not usable for one animal at 12 months and for another animal at 18 months. A repeated measures ANOVA on the number of days each rat required to entrain to the new light:dark schedule for the remaining animals (5 female and 9 male) from 12 to 18 months indicated an effect of age [*F*(2,12) = 40.6, *p* < 0.0001] in the absence of a sex difference. In this case, the number of days to entrain to the shift in light schedule was increased 2-fold for older animals (Figures 5A,B).

### Regression Analysis

**FIGURE 6:**
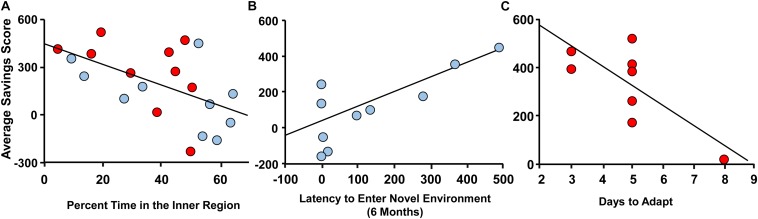
Correlates of variability in spatial memory at 12 months. Spatial memory at 12 months, measured as average savings scores, for males (blue circles) and females (red circles) plotted against **(A)** percent time in the inner region of the open field at 12 months, **(B)** latency to enter the novel environment at 6 months, and **(C)** days to adapt to a shift in light/dark cycle at 12 months.

Memory deficits began to emerge by 12 months. In order to provide predictors of memory impairment, averaged savings scores for the 12-month DMTP memory task were examined. For multiple regression analysis, sex was included as a relevant variable and the other variables were selected based on literature suggesting that age-related cognitive decline is associated with a decreasing level of activity (Shoji et al., 2016; Logan et al., 2018), altered response to novelty (Gallagher and Burwell, 1989; Dellu et al., 1994; Rowe et al., 1998; Collier et al., 2004; Dellu-Hagedorn et al., 2004), and circadian function (Issa et al., 1990; Winocur and Hasher, 1999; Gritton et al., 2012). Importantly, using all animals at 12 months, the averaged savings score was not different by sex (male = 116 ± 63; female = 265 ± 73, *p* = 0.14). The 12-month savings scores were not correlated with total one day 24 h wheel-running activity. However, 12 months savings scores correlated with response to novelty in the open field (*r* = 0.59, *p* = 0.027, 1−β = 0.80). In this case, sex was not a significant predictor, and memory was negatively associated with the percent time in the inner region (r = −0.55, *p* = 0.031, 1−β = 0.37; Figure 6A). No correlation was observed for memory and time to enter a novel environment at 12 months. A previous longitudinal study found that impaired spatial memory in aged rats was associated with high reactivity to novelty previously measured in adulthood (Dellu et al., 1994; Dellu-Hagedorn et al., 2004). Therefore, we performed multiple regression with the 12 months measures of DMTP memory and latency to enter the novel environment measured at 6 months. The results indicated a significant regression (*r* = 0.62, *p* = 0.018, 1−β = 0.85), which was due to sex (*p* = 0.01). When each sex was examined separately, only males exhibited a correlation with better memory associated with a longer delay to enter the novel arena (*r* = 0.79, *p* = 0.007, 1−β = 0.81; Figure 6B). Finally, savings scores tended to correlate with days to entrain to a shift in light schedule (*p* = 0.066). However, this was largely due to sex differences (*p* = 0.024) and a regression in each group indicated females (*n* = 8) with poorer memory exhibited more days to entrain to the shift in light/dark cycle (*r* = 0.74, *p* = 0.037, 1−β = 0.56; Figure 6C).

### Network Connectivity

Measures of functional connectivity were obtained following behavioral characterization at 12 months. Data for network metrics of whole brain functional connectivity were examined across all animals to look for differences associated with sex or cognitive function. No sex differences were observed in small worldness, clustering coefficient, path length, modularity or node strength (Supplementary Figure S1). To examine differences associated with behavioral performance, imaging data for all male and female rats were pooled. The savings scores recorded for the DMTP task at 12 months were averaged across all delays and a mean split of averaged savings scores was employed to classify animals as poor/impaired (*n* = 7 males, *n* = 3 females, average saving score of 7 ± 46) and best/unimpaired (*n* = 3 male, *n* = 7 female, average savings score of 370 ± 29). Again, no differences were observed in small worldness, clustering coefficient, path length, modularity or node strength (Supplementary Figure S2).

**FIGURE 7:**
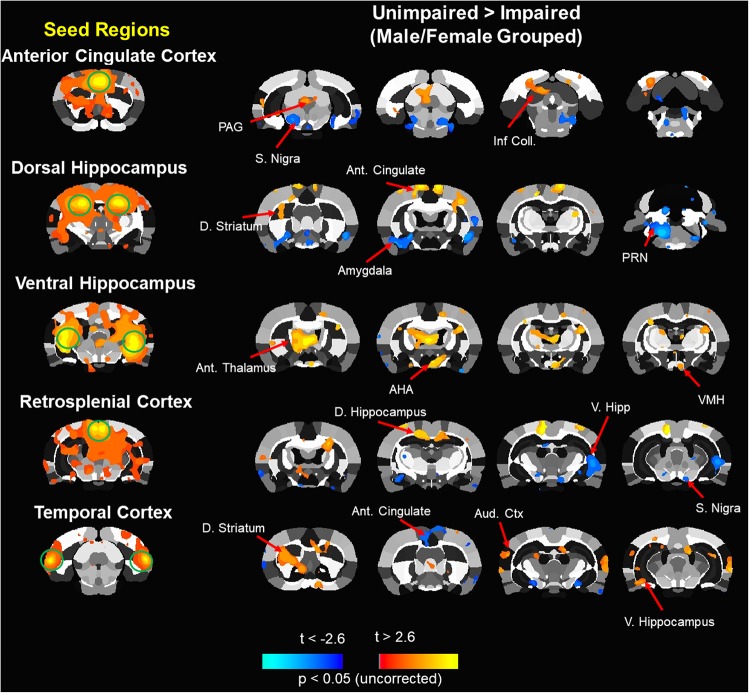
Composite resting state functional connectivity maps for impaired and unimpaired animals. Seed based functional connectivity analysis. Images processed for functional connectivity are overlaid onto a segmented atlas of the rat brain with a threshold of *p* < 0.05. Column on the left shows the seed regions. To the right, in each row, are maps indicating areas that showed a significant reduction in functional connectivity (blue) or increases in functional connectivity (red/yellow) in impaired vs. unimpaired. Ant, anterior; AHA, anterior hypothalamic area; D, dorsal; Inf. Coll, inferior colliculus; PAG, periaqueductal gray; PRN, pontine reticular nucleus; S. Nigra, substantia nigra; V, ventral; VMH, ventromedial hypothalamus.

Next, an ROI specific analysis was performed in which functional connectivity with a particular seed region was assessed comparing poor/impaired and best/unimpaired middle-age animals. Figure 7 illustrates the performance-based differences. In most cases, the seed region was associated with increased and decreased connectivity of impaired relative to unimpaired animals. For example, the retrosplenial cortex (RSC) of impaired animals exhibited increased and decreased connectivity with the dorsal and ventral hippocampus, respectively. Previous work indicates a major role for the RSC in episodic memory in older humans and spatial reference memory in aged rats (Vann et al., 2009; Ash et al., 2016; Kaboodvand et al., 2018). In addition, the dorsal hippocampus exhibited altered connectivity with other memory systems (McDonald and White, 1993), increasing connectivity with the dorsal striatum and decreasing connectivity with the amygdala. In contrast, the ventral hippocampus of impaired animals exhibited increased connectivity with central areas including the thalamus and hypothalamus.

## Discussion

The current study provides longitudinal analysis of age- and sex-related changes in physical, psychological, and cognitive function. In addition, we provide information on behavioral, physical, and biological correlates for the decline of spatial memory. Spatial episodic or spatial working memory, which becomes impaired around middle-age in rats and mice, is a delay-dependent memory for rapidly acquired and flexible spatial information (Foster, 2012). Tasks that involve flexible spatial information have an advantage for longitudinal studies in that these tests can be repeated over the course of aging. Previous longitudinal studies employing several different spatial episodic or spatial working memory tasks have identified age-related impairment in memory, with minimal carryover influences (Ando and Ohashi, 1991; Forster and Lal, 1992; Vallee et al., 1999; Hartman et al., 2001; Markowska and Savonenko, 2002; Dellu-Hagedorn et al., 2004; Sabolek et al., 2004).

In the current study, using the DMTP task, we confirmed an age-related impairment in retaining spatial memory with increasing delays, and the severity or propensity of impairment increased with advancing age. In contrast, novel location recognition was less sensitive in detecting age differences. Although the reason for the lack of sensitivity is unclear, memory performance on this task, including sex differences, is sensitive to stress (Luine, 2002; Bath et al., 2017) and delay interval (Sutcliffe et al., 2007; Wimmer et al., 2012). In general, individual variability in spatial memory on the DMTP task manifested in middle-age and impairment for delays greater than 1 min was obvious for most animals at 18 months; however, there was evidence for a sexually dimorphic trajectory of decline. The results are consistent with previous research on sex differences in episodic memory in humans (Herlitz et al., 1999; Dixon et al., 2004; Wahlin et al., 2006) and cross-sectional studies in rats, which indicate that middle-age females outperform males on a temporal episodic memory task (Rossetti et al., 2018), and aged females outperform males on a delayed match-to-location task (Lukoyanov et al., 1999). In addition, spatial memory for older females may benefit from previous cognitive testing as young adults (Talboom et al., 2014). In contrast, others have observed that, for a 3 min delay, older female rats (24 months) exhibited poorer performance on a repeated acquisition task (Markowska, 1999).

A sex difference was evident at 12 months when analysis was limited to animals that were healthy throughout the 18 months of testing. When all animals were included at 12 months, the averaged savings score was not different by sex. In this case, 7 of 10 males were below the group mean and most females, 7 of 10, exhibited superior spatial memory at 12 months. For the females that were below the mean, two were removed prior to 18 months due to health concerns. This suggests that undetected health problems may contribute to variability in cognitive function. Interestingly, health status of humans during aging may reduce sex differences by suppressing the superior episodic memory performance of females (Wahlin et al., 2006). Thus, future longitudinal studies should consider healthspan as a factor contributing to variability in memory function.

Repeated testing over the lifespan does not appear to influence sensory-motor function, such that the decline in locomotor activity is similar for longitudinal and cross-sectional studies (Altun et al., 2007). Longitudinal studies in humans indicate that sex and the level of physical activity are relevant factors in predicting cognitive decline (Olaya et al., 2017; Hamer et al., 2018); although, the direction of the temporal relationship between cognitive function and physical activity is not always clear (Stijntjes et al., 2017). Moreover, there is considerable evidence that exercise can protect, maintain, or rejuvenate the brain (Cui et al., 2009; Kumar et al., 2012; Speisman et al., 2013; Voss et al., 2013). Thus, the short-duration (24 h) aerobic exercise (activity wheel running), which preceded cognitive testing by approximately a week, may have influenced the brain and cognitive function (Cabral et al., 2019). However, it is important to emphasize that we did not control the level of wheel running. Furthermore, memory was not correlated with the level of wheel running activity at the 12 months testing point. Rather, the wheel running was voluntary and likely related to the general level of activity. It has been suggested that for rodents activity and cognition function decline in parallel (Shoji et al., 2016; Logan et al., 2018). Moreover, female rodents are more active than males (Stowie and Glass, 2015; Rosenfeld, 2017) and cognition may be differentially influenced by exercise (Barha et al., 2017). We confirmed heightened activity of females as increased wheel running during a 24 h period. Interestingly, females exhibited a marked decrease in wheel-running activity and cognitive function from 12 months to 18 months, during the time when rodents undergo estropause. Indeed, estradiol treatment of older animals promotes wheel-running activity (Stern and Murphy, 1972; Gerall et al., 1973; Gentry and Wade, 1976) and episodic memory (Yonker et al., 2006; Bean et al., 2015). Thus, the decline in ovarian estradiol levels in midlife, as a mechanism determining memory function, represents an important opportunity of examination in future longitudinal studies (Rentz et al., 2017).

Response to novelty has been used as a measure of anxiety and neophobia. Individual differences in response to novelty may predict the propensity for drug abuse and memory function (Antoniou et al., 2008; Flagel et al., 2014). In the current study, time spent in the inner region of an open field and time to enter a novel environment were employed as measures of reactivity to novelty. For the open field task, aging was associated with an increase in time exploring the center of the open field. Cross-sectional studies examining exploration on the open field task indicate that exploration is decreased, increased, or not changed with age (Miyagawa et al., 1998; Boguszewski and Zagrodzka, 2002; Torras-Garcia et al., 2005; Bergado et al., 2011; Meyza et al., 2011; Moretti et al., 2011; Shoji and Miyakawa, 2019). The increased exploration of the inner region, as well as the age-related decrease in time to enter a new environment observed in the current study, may be due to the extensive handling and repeated testing across a number of environments (Hall et al., 1997). In general, environmental enrichment is associated with decreased anxiety (Fox et al., 2006), which can be observed in older animals (Galani et al., 2007; Leal-Galicia et al., 2008; Hughes and Collins, 2010; Sampedro-Piquero et al., 2014).

Interestingly, response to novelty measures were correlated with memory and the relationship was opposite to that usually observed in cross-sectional studies. Despite the increased time in the inner region of the open field with age, animals with better memory scores spent less time in the inner region. Cross-sectional studies indicate that impaired acquisition of a spatial reference memory in older cohorts is associated with heightened neophobia, including decreased activity in the open field (Gallagher and Burwell, 1989; Rowe et al., 1998; Collier et al., 2004). In contrast, the time to enter a new environment, a possible measure of neophobia, did not correlate with impaired spatial memory at 12 months of age. Several important procedural differences likely underlie the outcome differences. The current study examined a form of spatial memory, which begins to decline in middle-age, whereas the previous studies examined an inability to acquire a spatial reference memory in older animals (Foster, 2012). Moreover, longitudinal and cross-sectional studies may produce different effects on anxiety and response to novelty. For studies that involve multiple age cohorts, the oldest animals may experience prolonged exposure to an impoverished environment, which can impair cognition (Winocur, 1998; Bell et al., 2009; Diniz et al., 2010; Volkers and Scherder, 2011; Sampedro-Piquero et al., 2014; Diamond, 2018; Sparling et al., 2018; Wang et al., 2018) and increase neophobia/anxiety (Hall et al., 1997; Hellemans et al., 2004). Together, the results highlight how cross-sectional and longitudinal studies may produce differences in the response to novelty.

Previous longitudinal studies have indicated that behavioral/cognitive measures in young adults (Dellu et al., 1994; Dellu-Hagedorn et al., 2004; Talboom et al., 2014; Hullinger and Burger, 2015) or middle-age (Stone et al., 1997) may predict future learning and memory performance. We observed that the differential response to enter a novel environment at 6 months predicted impaired spatial memory in males at 12 months. This result is similar to studies that find a higher-level response to novelty, generally observed as increased motor activity, predicts an increased propensity for drug abuse and cognitive impairment (Antoniou et al., 2008; Flagel et al., 2014). In particular, a longitudinal study in rats observed that impaired spatial memory of older animals was associated with differential reactivity to novelty measured earlier, from 2 to 16 months, with poorer performance in animals originally characterized as high responders (Dellu et al., 1994; Dellu-Hagedorn et al., 2004). Moreover, similar to the current study, reactivity to novelty declined over age with repeated testing. Together, the results suggest that for longitudinal studies, reactivity to novelty decreases with age due to repeated testing. Furthermore, those animals that initially display low reactivity to novelty exhibit better memory as they age.

The mechanism that links the increased response to novelty (locomotion) with impaired cognition is unclear. Possible mechanisms include impaired spatial learning, resulting in poorer habituation to exploration (Soibam et al., 2013). In addition, high responders may have an elevated stress response (Piazza et al., 1991; Kabbaj et al., 2000), which could affect the hippocampus and memory (Magalhaes et al., 2019). Alternatively, disruption of circadian rhythms can also influence memory and the response to a novel environment (Karatsoreos et al., 2011; Kondratova et al., 2010). Circadian rhythms persist across numerous physiological processes. For instance, there are circadian patterns of body temperature, activity-wakefulness, locomotor activity patterns, and drinking behavior, and each of these processes exhibit age-related changes in rodents (Weinert, 2000). Aging also reduces tissue-specific re-entrainment following an acute phase shift (i.e., scientific jet-lag (Sellix et al., 2012). Consistent with these previous investigations, we report an age-related increase in the number of days required for behavioral re-entrainment following an acute phase advance. Furthermore, poorer memory was correlated with an increased number of days to adapt to a change in light cycle at 12 months in females. Females may be more sensitive to the effects of circadian disruption on cognition (Santhi et al., 2016); however, further exploration is needed to identify the specific relationship between loss of circadian robustness and cognition during aging.

We observed both increased and decreased connectivity associated with impaired spatial memory. Altered connectivity may represent an underlying mechanism for impairment or compensation for impaired memory. For example, the dorsal hippocampus of impaired animals exhibited altered connectivity with different memory systems involving the striatum and amygdala (McDonald and White, 1993). Increased connectivity of the hippocampus and striatum could represent compensation for impaired spatial memory. Interestingly, the dorsal hippocampus of the rat corresponds to the posterior hippocampus in primates, and in older humans, the posterior hippocampus exhibits a decrease in functional connectivity with regions involved in episodic memory (Damoiseaux et al., 2016) and increased structural connectivity with the dorsal striatum (Liu et al., 2017). We observed an increase in connectivity between the ventral hippocampus and more central regions, including the thalamus, which is consistent with human studies demonstrating this increased functional connectivity in association with greater impairment in visual-spatial memory (Goldstone et al., 2018).

The RSC in memory-impaired animals exhibited increased and decreased connectivity with the dorsal and ventral hippocampus, respectively. In humans, an increase in functional connectivity, which is initially associated with memory impairment, may predict further cognitive decline, reduced functional connectivity, and loss of brain volume with more advanced age (Reuter-Lorenz and Cappell, 2008; Wang et al., 2011; Staffaroni et al., 2018; Zheng et al., 2018). Similarly, in rodent models, impaired spatial episodic memory in middle-age may predict more severe cognitive deficits with advancing age (Foster, 2012) and increased dorsal hippocampus-RSC connectivity may foretell a future loss of hippocampus-RSC connectivity (Ash et al., 2016; Parent et al., 2017), and loss of hippocampal volume (Magalhaes et al., 2017; Reichel et al., 2017). For example, in a rat model of Alzheimer’s disease, higher dorsal hippocampal connectivity in middle-age progressed to reduced dorsal hippocampus-RSC connectivity with age (Parent et al., 2017). Similarly, older rats characterized as impaired on a spatial reference memory task exhibited decreased dorsal hippocampus-RSC connectivity (Ash et al., 2016). Finally, memory impairment in middle-age mice predicts accelerated dorsal hippocampal volume loss during aging (Reichel et al., 2017). While increased dorsal hippocampus-RSC connectivity in conjunction with impaired memory is consistent as an early biomarker of cognitive decline, future studies should determine if the differences in connectivity are linked to the response to stress/novelty in adulthood (Magalhaes et al., 2017), as well as a decrease of connectivity/volume and more severe hippocampal memory deficits with advanced age. These parallels between human and rodent resting state networks hold promise for the use of functional connectivity to track age-associated cognitive network changes. However, it is important to note that most rodent studies use anesthesia protocols to minimize movement and physiological variations across subjects during scanning. Therefore, it is important to consider the use of anesthesia during scanning when interpreting fMRI results in rodents. The levels of isoflurane used in the present study are below levels that reduce selectivity of spontaneous BOLD activity (e.g., >1.8%), and has been applied in numerous imaging experiments (Liu et al., 2013; Jonckers et al., 2014).

The current study confirms the results of longitudinal studies, which indicate that individual variability in memory is observed in middle-age. While few studies have longitudinally examined sex differences in cognition, the results support cross-sectional studies that indicate sexual dimorphism in cognitive decline. The results suggest that future longitudinal studies should consider healthspan, circadian function, voluntary activity levels, and estradiol levels in contributing to sex differences in the trajectory of cognitive decline. Furthermore, comparison of the results with cross-sectional studies highlights differences in the response to novelty, likely due to differences in environmental stimulation/impoverishment. Moreover, for animals that are repeatedly tested, variability in response to novelty appears to predict cognitive function, with high responders exhibiting earlier cognitive decline, particularly for males. The neural and hormonal mechanisms that link response to novelty with individual differences in cognitive decline remain to be determined. Finally, spatial memory impairment in middle-age was associated with altered functional connectivity. Future studies will be required to determine if differences in spatial episodic or working memory and functional connectivity in middle-age can predict future changes in connectivity, loss of brain volume, and impaired spatial reference memory. In addition, it will be interesting to see if environmental enrichment or exercise, which are thought to contribute to brain maintenance and neural reserve, will influence the relationship between connectivity and cognition.

## Data Availability Statement

The datasets generated for this study are available on request to the corresponding author.

## Ethics Statement

The animal study was reviewed and approved by the Institutional Animal Care and Use Committee at the University of Florida.

## Author Contributions

AR, BY, JB, and AK collected the behavior data. MF collected and analyzed brain imaging data. CW and KE collected and analyzed circadian adaptation data. TF designed experiments, analyzed data, constructed figures, and wrote the manuscript.

## Conflict of Interest

The authors declare that the research was conducted in the absence of any commercial or financial relationships that could be construed as a potential conflict of interest.

## Footnotes

1 www.itksnap.org

## Supplementary Material

FIGURE S1 The top panels illustrate the three-dimensional functional connectivity maps of females (*n* = 10) and male (*n* = 10) rats. The lower graphs illustrate no sex difference in global network connectivity. (A) Symbols represent mean (±SEM) for small world coefficient (left), clustering coefficient (center), and path length (right) over the graph density thresholds for females (filled circle) and male (open square) animals.

FIGURE S2 A mean split of the 12-month average savings scores was used to classify animals as impaired and unimpaired. The top panels illustrate the three-dimensional functional connectivity maps of the impaired (left) and unimpaired (right) performers. The lower graphs illustrate no difference in global network connectivity associated with cognitive function at 12 months. (A) Symbols represent mean (±SEM) for small world coefficient (left), clustering coefficient (center), and path length (right) over the graph density thresholds for impaired (filled circle) and unimpaired (open square) animals.
