# Supplementary figures and images for: Longitudinal Characterization and Biomarkers of Age and Sex Differences in the Decline of Spatial Memory
Source: Front Aging Neurosci. 2020 Feb 20;12:34. doi: 10.3389/fnagi.2020.00034 (PMC7044155; doi:10.3389/fnagi.2020.00034)

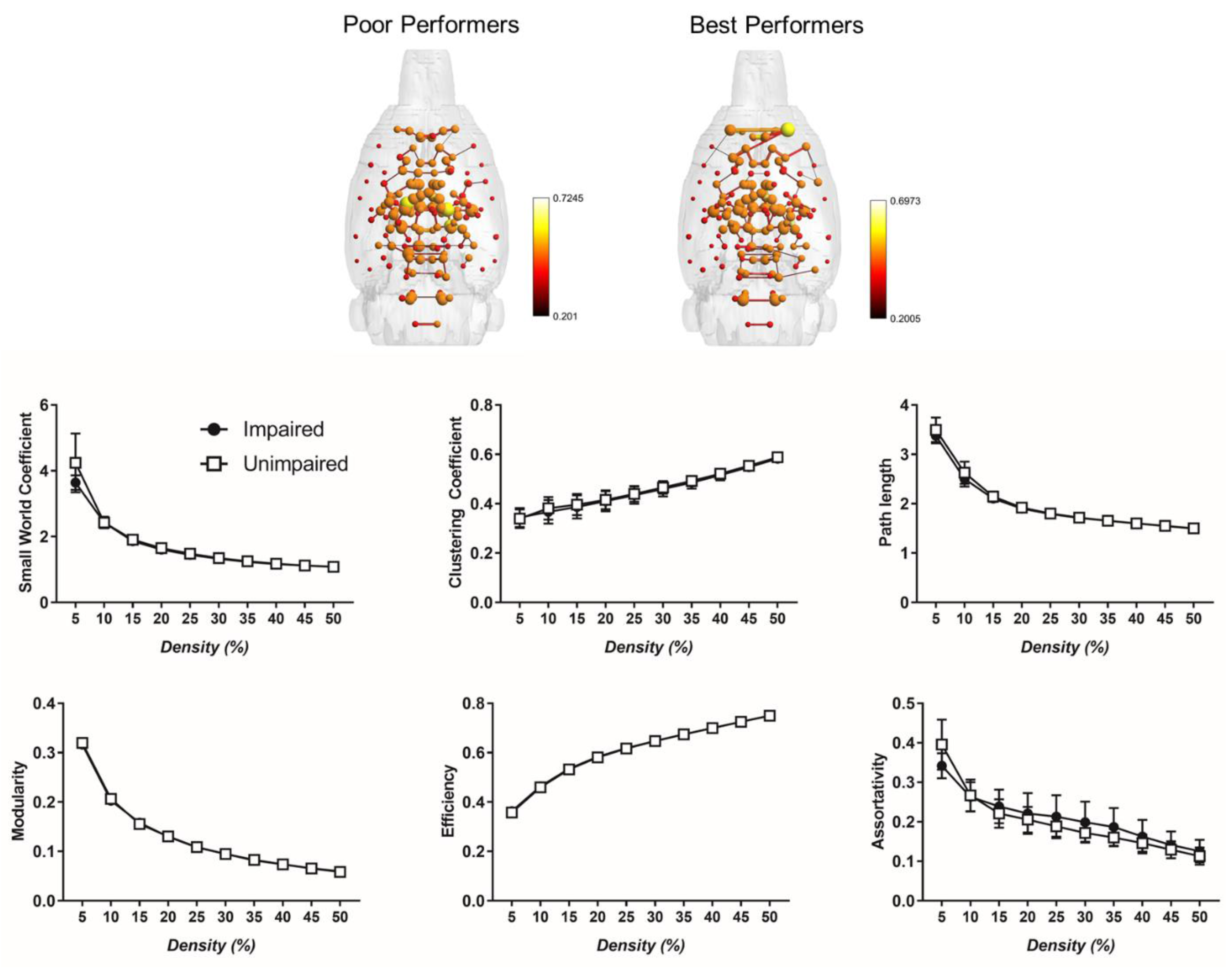

Supplement: FIGURE S1 — The top panels illustrate the three-dimensional functional connectivity maps of females (n = 10) and male (n = 10) rats. The lower graphs illustrate no sex difference in global network connectivity. (A) Symbols represent mean (±SEM) for small world coefficient (left), clustering coefficient (center), and path length (right) over the graph density thresholds for females (filled circle) and male (open square) animals. [file Image_1.TIF]

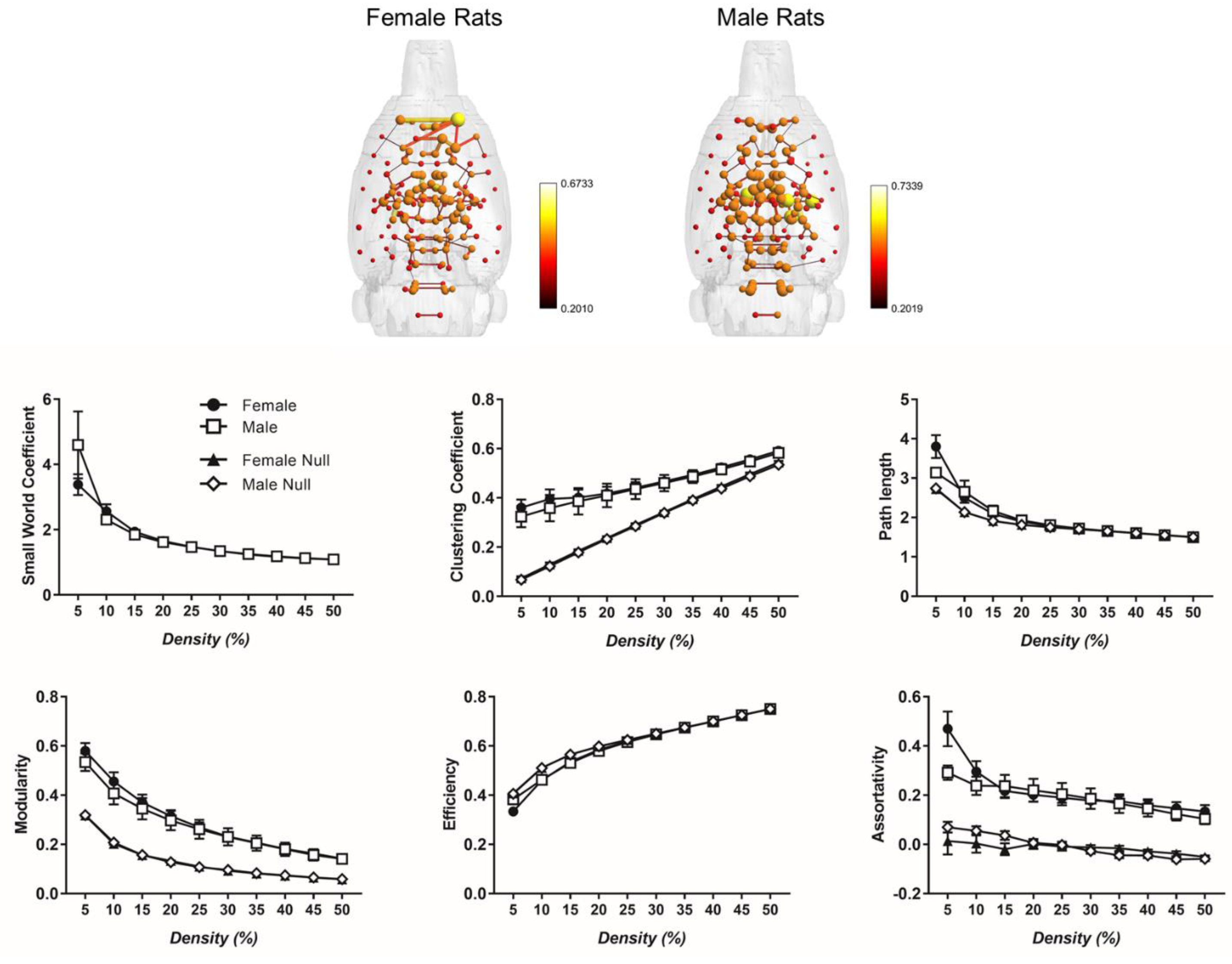

Supplement: FIGURE S2 — A mean split of the 12-month average savings scores was used to classify animals as impaired and unimpaired. The top panels illustrate the three-dimensional functional connectivity maps of the impaired (left) and unimpaired (right) performers. The lower graphs illustrate no difference in global network connectivity associated with cognitive function at 12 months. (A) Symbols represent mean (±SEM) for small world coefficient (left), clustering coefficient (center), and path length (right) over the graph density thresholds for impaired (filled circle) and unimpaired (open square) animals. [file Image_2.TIF]
